# IL-23 and IL-27 Levels in Macrophages Collected from Peripheral Blood of Patients with Healing Vs Non-Healing Form of Cutaneous Leishmaniasis

**Published:** 2012

**Authors:** S Tolouei, K Ghaedi, A Khamesipour, M Akbari, M Baghaei, SJ Hasheminia, M Narimani, SH Hejazi

**Affiliations:** 1 Department of Parasitology and Mycology, School of Medicine, Isfahan University of Medical Sciences, Isfahan, Iran; 2 Department of Biology, School of Sciences, University of Isfahan, Department of Cell and Molecular Biology, Royan Institute for Animal Biotechnology, ACECR, Isfahan, Iran; 3 Center for Research and Training in Skin Diseases and Leprosy, Tehran University of Medical Sciences, Tehran, Iran; 4 School of Medicine, Isfahan University of Medical Sciences, Isfahan, Iran; 5 Department of Biology, Faculty of Basic Sciences, Islamic Azad University Shahrekord Branch, Shahrekord, Iran; 6 Department of Immunology, School of Medicine, Isfahan University of Medical Sciences, Isfahan, Iran; 7 Skin Disease and Leishmaniasis Research Center, Department of Parasitology and Mycology, School of Medicine, Isfahan University of Medical Sciences, Isfahan, Iran

**Keywords:** IL-23, IL-27, Macrophages, Promastigote, Cutaneous leishmaniasis

## Abstract

**Background:**

In this study the level of IL-23 and IL-27 produced by macrophages derived from peripheral blood mononuclear cell culture collected from patients with healing or non-healing form of cutaneous leishmaniasis lesion were compared before and after treatment with live *Leishmania* to explore whether IL-23 or IL-27 plays any role in healing process of cutaneous lesions induced by *L. major*.

**Methods:**

Twenty patients resident in Isfahan Province, with healing or non-healing form of cutaneous leishmaniasis lesion caused by *Leishmania major* participated in this study. *In vitro* productions of IL-23 and IL-27 by peripheral blood derived macrophages, before and after stimulation with live *L. major* (MRHO/IR/75/ER) promastigotes were evaluated using ELISA method****. Patient with healing form of lesion received no treatment and patient with non-healing form of lesion received at least 2 courses of glucantime.

**Results:**

The mean production of IL-23 and IL-27 from macrophages of patients with healing form of lesion was significantly higher than patients with non-healing form of lesion. The levels of IL-23 and IL-27 in culture supernatants before and after stimulation in healing form of CL was significantly higher than non- healing form of CL (*P <* 0.001).

**Conclusion:**

IL-23 and IL-27 might play a role in human leishmaniasis and further studies are needed to understand the role of IL-23 and IL-27 in leishmaniasis.

## Introduction

Cutaneous leishmaniasis (CL) is a self-healing lesion which heals spontaneously but rarely might develop to a non-healing form of disease refractory to various types of remedies (1). It seems that clinical manifestation of leishmaniasis depends upon the type of immune response generated and the species of *Leishmania* (2, 3). Although tremendous data is available in murine model of leishmaniasis which indicate generation of Th1 type of response in healing form of disease and Th2 type of response for progressive infection (4–7) but immunological surrogate marker(s) of healing and protection in human is yet not well defined (8–11). Studies on various cytokine profile of Th1/Th2 are published with controversy results (8–12). Cytokines have fundamental roles in development and regulation of immune responses against various infectious and non-infectious diseases (13–16). Recently, IL-23 and IL-27 were identified as heterodimeric cytokines which are structurally and functionally related to IL-12 family which promote IFN-γ production and Th1 development (17, 18). IL-12 composed of two subunits: p35 and p40, and IL-23 consisted of the p40 subunit of IL-12 and p19 subunit (19). IL-27 is another heterodimeric cytokine composed of a p40-related molecule, and p28 (20). There is evidence that IL-27 promotes Th1 differentiation and IL-23 plays an important role in proliferation of memory-type Th1 cells. IL-23 and IL-27 are involved in the regulation of Th17 response (21), while IL-23 induces differentiation of Th17 cells; IL-27 inhibits Th17 differentiation and IL-17 production by GATA-3 expression through the Stat1-dependent pathway (22). As it is well established that successful control of infection against intracellular pathogens requires the production of IFN-γ, thus presence of IL-12 family as an inducer of Th1 phenotype is important (17, 23).

In this study the level of IL-23 and IL-27 produced by macrophages derived from peripheral blood mononuclear cell culture collected from patients with healing or non-healing form of lesion were compared before and after treatment with live *Leishmania* to explore whether IL-23 or IL-27 plays any role in healing process of cutaneous lesions induced by *L. major*.

## Materials and Methods

This study was approved by the Ethical Committee on Human Research, Isfahan University of Medical Sciences. The volunteers were informed about the objective and procedure of the study and those who were willing to participate, donate blood sample and sign an informed consent were recruited.

### Study groups

Two groups of CL patients were selected from the patients referred to the Skin Disease and CL Research Center, Isfahan University of Medical Sciences.

Ten parasitologically proven CL patients with healing form of lesion with onset less than 6 months and no history of treatment for CL and 10 parasitologically proven CL patients with non-healing form of lesion with duration of lesion more than 1 year and history of at least 2 courses of Glucantime treatment were included in this study. Diagnosis was based on observation of *Leishmania* using Giemsa stained smear and/or growth of promastigotes in NNN culture. Identification of *Leishmania* causative agent of the lesions was done using PCR method.

### DNA extraction

In order to identify the *Leishmania* species, PCR method was used. The promastigotes isolated from the culture of each patient's lesion were centrifuged at 700×g, 4°C for 10min, and then were washed 3 times with PBS. DNA was extracted using High Pure PCR Template Preparation Kit (Roche, Germany). Purified DNA was eluted in 200µl of elution buffer and stored in −20°C until use. DNA from *L. major* (MRHO/IR/75/ER) was used as a control.

### PCR amplification

Specific oligonucleotide primer was designed based on GenBank accession no. AJ300485, (Forward: CAA CAC GCC GCC TCC TCT CT, Reverse: CCT CTC TTT TTT CGC TGT GC) (Bioneer). Amplifications were performed in 25 µl containing, 2.5µl PCR buffer(10xbuffer), 0.75mM MgCl_2_ (50mM), 0.5 mM dNTP (10mM), 5pmol of each primer, 0.25unit of Taq polymerase (5u/ µl) and 1µl of DNA template. DNA was amplified using thermal cycler (Corbett) under the following conditions: at 94°C for 5min followed by 25 repetitive cycles of 30s at 94°C, 30s at 54°C, 30s at 72 °C and a final elongation at 72°C for 10min.

PCR products were run on 1.5% agarose gel in TBE buffer. The bands were visualized under UV illumination and the size of the products was determined.

### Isolation of mononuclear cells

Peripheral Blood Mononuclear Cells (PBMCs) were isolated using Ficoll-hypaque density gradient (Lymphodex, Germany). PBMCs were washed 3 times with PBS and the pellet was resuspended in 4 ml of RPMI 1640 medium (Gibco, Germany), supplemented with 2mM L-glutamine, penicillin (100u/ml), gentamicin (100µg/ml) and 10% heat-inactivated Fetal Bovine Serum (Gibco). The cell number was estimated using light microscopy and the viability of the cells was checked by trypan blue (0.4%). PBMCs were distributed in two 3.5 cm tissue culture plates with a concentration of 5x10^6^ cell/ml in each plate and incubated at 37°C with 5% CO_2_ for 2 hours and then non adherent cells were removed by washing with PBS. Adherent cells were incubated at 37°C with 5% CO_2_ for additional 6 days in order to differentiate to macrophages (24) and the medium was changed every 72h. The size of the cells was morphologically increased and numerous short pseudopods were formed. The cellular phenotype was analyzed by flowcytometry using anti CD14 monoclonal antibody (Abcam, United Kingdom) (data not shown).

### Parasite

*L. major* (MRHO/IR/75/ER), was used for leishmanization and preparation of experimental *Leishmania* vaccine and leishmanin was used in this experiment (25). Parasite was grown on biphasic NNN medium and sub passaged in RPMI 1640 supplemented with 10% FBS. Promastigotes were harvested at stationary phase.

### Macrophage and parasite interaction

After 6 days monocyte derived macrophages were infected with *L. major* promastigotes harvested at stationary phase at a ratio of 5:1 parasite/macrophage. After 6h, non-internalized free promastigotes were removed by washing. Then, the infected cells were incubated in complete RPMI 1640 for additional 18h. The culture supernatants were collected and stored at -70°C for IL-23 and IL-27 titration using ELISA assay.

### Cytokines measurement

The frozen culture supernatants were thawed and cytokine levels of IL-23 and IL-27 were determined using commercial kits (Abcam, United Kingdom) according to the manufacturer's instructions. Briefly during the first incubation IL-23or IL-27 antigen was added to the wells. After washing, biotinylated monoclonal antibody specific for IL-23or IL-27 was incubated. Then the enzyme (streptavidin-peroxydase) was added. After incubation and washing to remove all unbounded enzyme, a substrate solution which acts on the bound enzyme was added. The absorbance of each well was read at 450nm. The concentration of IL-23 and IL-27 in the treated and untreated supernatants was determined using standard curve. Cytokine values were expressed as picogram/ml (pg/ml).

### Statistical analysis

Statistical analysis was performed using SPSS version 16. Student's paired and independent *t* test were used to determine whether significant difference exists between the cytokine levels.

## Results

Among 20 patients recruited with proven CL, 18 were male and 2 were female. The basic information of the study is summarized in Table 1.

**Table 1:** Major characteristics of study groups

Characteristic	Study group	
	Healing	Non-healing
No	10	10
Age(range)/ years	18-60	22-65
M/F ratio	8/2	10/0
Average duration of lesion	3.1 month	2.3 year

### Molecular analysis

**Fig. 1:**
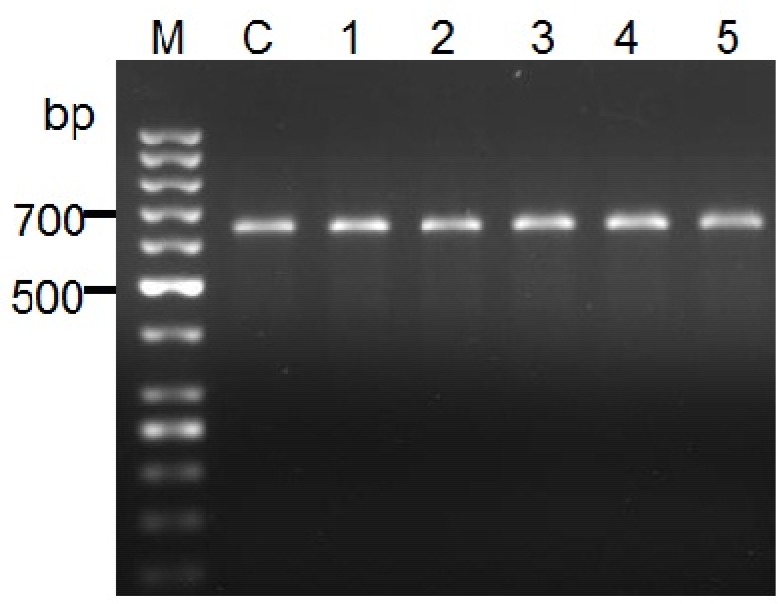
PCR product of the internal transcribed spacer1 (ITS1) region of genomic DNA samples from healing and non-healing patients’ lesions. DNA from *L.major* (MRHO/IR/75/ER) used as control (C). No.1, 2 3 are samples from healing patients and No.4, 5 are obtained from non-healing individuals. M, is DNA ladder 50 bp (Fermentas)

All samples were undertaken microscopic examinations and PCR assay. PCR experiment revealed a single 625bp DNA fragment was seen on the gel which is accordance with DNA fragment of *L. major* reference (Fig. 1).

### Cytokine production

#### IL-23 levels

The level of IL-23 in healing and non-healing form of CL before stimulation was 182.9±23.9 pg/ml and 141.3±13.5 pg/ml respectively which was significantly different (*P <* 0.001). The level of IL-23 after stimulation in supernatants of macrophages in healing and non-healing form of CL was 386 ±43.9 pg/ml *vs*. 235 ±43.9 pg/ml which was significantly different (*P <* 0.001). The level of IL-23 before and after stimulation in supernatants of macrophages in healing form of CL was 182.9±23.9pg/ml and 386.9±43.9 pg/ml, respectively, whereas the level of IL-23 in non-healing form was 141.3±13.5pg/ml and 235.5±43.9pg/ml, respectively. The differences between IL-23 level in supernatant of the macrophage culture before and after stimulation in patients with healing and non-healing form of CL were 203.9±38.3 *vs*. 94.1±36.5 respectively.The data were shown the difference in healing form of CL was significantly higher than non-healing form of CL (*P <* 0.001).

**Fig. 2:**
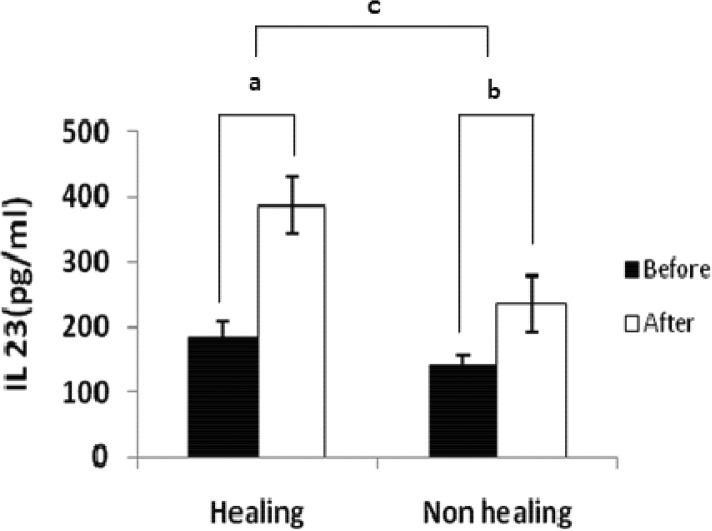
Cytokine production from macrophages in patients’ with healing and non healing lesions. Macrophages were stimulated with or without live *L. major* promastigotes. After 24 hours supernatants were assayed for IL-23 production. Results were obtained by an ELISA assay. Difference between cytokine production in stimulated and unstimulated macrophages in healing group (a), difference between cytokine production in stimulated and unstimulated macrophages in non- healing group(b)and difference between a and b(c). Data are expressed as the mean±standard deviation of the mean. Significant differences were shown between these groups (*P* < 0.001)

**Fig. 3:**
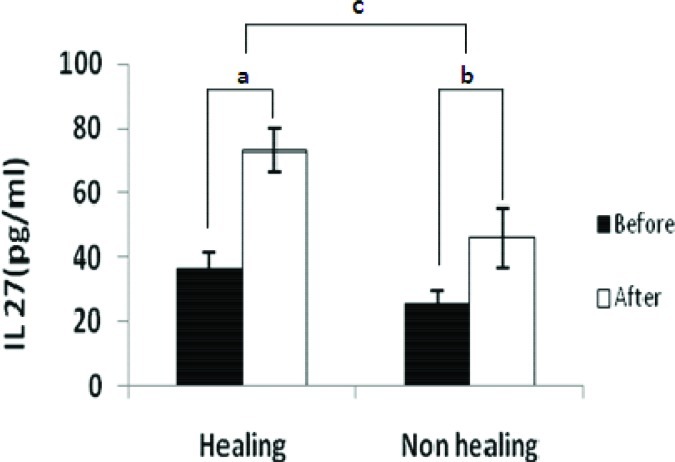
Cytokine production from Macrophages in patients’ with healing and non healing lesions. Macrophages were stimulated with or without live *L. major* promastigotes. After 24hours supernatants were assayed for IL-27 production. Results were obtained by an ELISA assay. Difference between cytokine production in stimulated and unstimulated macrophages in healing group (a), difference between cytokine production in stimulated and unstimulated macrophages in non- healing group(b)and difference between a and b(c). Data are expressed as the mean ± standard deviation of the mean. Significant differences were shown between these groups (*P* < 0.001)

#### IL-27 level

The concentration of IL-27 in healing and non-healing form of CL before stimulation was 36.6±4.5 pg/ml and 25.4±4.2 pg/ml respectively which was significantly different (*P <* 0.001). The level of IL-27 after stimulation in supernatants of macrophages in healing and non-healing form of CL was 73.3 ±6.8 pg/ml *vs*. 46.2±9.2 pg/ml which was significantly different (*P <* 0.001). The concentration of IL-27 before and after stimulation in supernatants of macrophages in healing form of CL was 36.6±4.5pg/ml and 73.3±6.8pg/ml, respectively, whereas the level of IL-27 in non-healing form was 25.4±4.2pg/ml *vs*. 46.2±9.2pg/ml. There was a significant difference (*P <* 0.001) in IL-27 level before and after stimulation in healing and non-healing form of CL.

The differences between IL-27 concentration in supernatant of the macrophage culture before and after stimulation in patients with healing form and non-healing form of CL was 36.7±6.48pg/ml vs. 20.8±7.78pg/ml respectively. The data were shown the difference of IL-27 in healing form of CL was significantly higher than non- healing form of CL (*P <* 0.001).

## Discussion

Although immune responses in leishmaniasis have been studied but still no surrogate marker(s) of healing and protection in human is identified (8-12). IL-12, IL-23 and IL-27 are related to the family which stimulates Th1 type of immune response. Effective immune response in intracellular parasites such as *Trypanosoma cruzi*, *Toxoplasma gondii* and *Leishmania major* is Th1 type of response (11, 26). In this study the level of IL-23 and IL-27 were studied to explore possible relationship between IL-23 and IL-27 and healing process in patients with CL caused by *L. major*. The results showed that the level of IL-23 and IL-27 in both groups of patient were elevated after the macrophages were stimulated with *L. major*. Moreover, the difference of IL- 23 and IL- 27 levels before and after stimulation was significantly (*P*<0.001) different between macrophages collected from patients with healing and non-healing form of the lesion. The differences was seen in IL-23 and IL-27 production in these two groups might be due to different interaction between the structures referred to pathogen-associated molecular patterns (PAMPs) that are recognized by pathogen recognition receptors (PRRs) of innate immune system (27). Another possibility is that different intracellular signaling pathway or adaptor molecules might be involved (28). This alteration may result from a difference in regulatory factors, enhancers and inhibitor molecules due to primary expression of cytokines (29). In the past few years immune responses against *Leishmania* infection and cytokine profile in human and animal leishmaniasis have been deeply studied (7, 8, 10, 11, 30). It is reported that IL-27 showed in vitro capacity to inhibit Th2 cell differentiation (31) and in another study it is shown that daily in vivo treatment of *Leishmania major* infected BALB/c mice by IL-27 induced lower parasite burden via up regulation of Th1 responses (22). It also shown that although IL-27 and its receptor are promoters of Th1 differentiation, but new finding showed that IL-27R is not required for the generation of IFN-γ mediated immunity to intracellular pathogens such as *Toxoplasma* infection (32). Almost all studies indicated that IL-12, plays a major role in resistance against toxoplasmosis but in the absence of IL-12, IL-23 can provide a limited mechanism of resistance to this infection (33). On the other hand, it is shown that IL-23 plays a key role in *Helicobacter hepaticus*–induced T cell–dependent colitis (34). It is also reported that Th1 development induced by Gram negative bacteria-primed macrophages is likely to be mediated by the joint action of different IL-12 family members and Th1 polarization might be driven by the action of the novel IL-12 family members IL-27 and/or IL-23 (35).

Taken together, seems that IL-23 and IL-27 may play a possible and complementary role with Th1 cytokines in human protection against *Leishmania* infection. Further studies are required to establish the role of these cytokines as surrogate markers in healing process of cutaneous leishmaniasis.
